# Traditional Medicine and Childcare in Western Africa: Mothers’ Knowledge, Folk Illnesses, and Patterns of Healthcare-Seeking Behavior

**DOI:** 10.1371/journal.pone.0105972

**Published:** 2014-08-22

**Authors:** Alexandra M. Towns, Sandra Mengue Eyi, Tinde van Andel

**Affiliations:** 1 Naturalis Biodiversity Center, Leiden University, Leiden, the Netherlands; 2 Le Centre National de la Recherche Scientifique et Technologique, Libreville, Gabon; Cairo University, Egypt

## Abstract

**Background:**

In spite of the strong role of traditional medicine in childcare in the pluralistic healthcare system in Western Africa, little information is known on mothers’ domestic plant knowledge. Identifying local perspectives and treatments of children’s illnesses, including folk illnesses, is essential to having a comprehensive understanding of how mothers make healthcare treatment decisions. We aimed to identify which infant illnesses Beninese and Gabonese mothers knew to treat with medicinal plants and for which illnesses they sought biomedical care or traditional healers.

**Methods:**

We conducted 81 questionnaires with mothers in Bénin and Gabon and made 800 botanical specimens of cited medicinal plants. We calculated the number of species cited per illness and the proportion of participants knowledgeable on at least one herbal remedy per illness. Using qualitative data, we described folk illnesses in each country and summarized responses on preferences for each of the three healthcare options.

**Results:**

Participants from both countries were most knowledgeable on plants to treat respiratory illnesses, malaria, diarrhea, and intestinal ailments. Mothers also frequently mentioned the use of plants to encourage children to *walk early*, monitor the closure of *fontanels,* and apply herbal enemas. Major folk illnesses were *atita* and *ka* in Bénin and *la rate* and *fesses rouges* in Gabon. Traditional healers were reported to have specialized knowledge of cultural bound illnesses. Malaria was frequently cited as an illness for which mothers would directly seek biomedical treatment.

**Conclusion:**

Mothers largely saw the three systems as complementary, seamlessly switching between different healing options until a remedy was found. Folk illnesses were found to give insight into local treatments and may reveal important neglected diseases. Due to high reported levels of knowledge on treating top statistical causes of infant mortality and folk illnesses, mothers’ medicinal plant knowledge should be included in the analysis of healthcare-seeking behavior for childcare.

## Introduction

Sub-Saharan African healthcare is essentially pluralistic, structured around three main systems: biomedical care, traditional healers, and popular knowledge [1]–[2]. In spite of the promotion of biomedicine by international healthcare organizations, traditional medicine remains the primary form of healthcare for more than 80% of African populations [3]. Traditional medical systems include not only traditional healers, but also the popular knowledge of local populations, known as domestic medicine or home remedies. Most ethnobotanical literature on traditional medicine is concentrated on the knowledge of traditional healers and largely overlooks domestic medicine, the knowledge of women [4], and more specifically, the knowledge of mothers [5]–[6]. Since home remedies (self-treatment with herbs) comprise the majority of African medicine [1], [7]–[8], domestic knowledge needs to be prioritized in medical research and reinforced in order to improve healthcare and enhance local populations’ responses to illness. This point is especially critical in high priority health populations, such as infants and children in sub-Saharan Africa [9].

African mothers’ knowledge of health is directly associated with children’s well-being, as women are largely responsible for childcare [8], [10]. Recent ethnobotanical research has found that mothers’ knowledge of herbal medicine has a positive effect on child health outcomes, including a decrease in infections [10]–[11]. Mothers who had high levels of plant knowledge and use have been shown to have healthier children [6] and a greater likelihood to take ill children to a dispensary, suggesting that knowledge in one healthcare domain corresponds with better overall understanding of health [10].

In spite of these correlations, biomedical studies have largely measured mother’s health-seeking behavior on factors related to biomedical care, such as formal education, distance to provider, and cost of obtaining care [12]. This literature overlooks if and what role local concepts of illness have in treatment choices and results in the loss of incorporating this information into infant health programs [13]. Local concepts of illness include not only local names, perceptions, and symptoms of biomedical illnesses, but also *cultural bound syndromes*, “a group of folk illnesses, each of which is unique to a particular group of people, cultural, or geographical area” [14]. Some scholars have cautioned that the “cultural” component of the term *cultural bound syndromes* emphasizes the biomedical perspective that biological illnesses are more objective than folk illnesses [14]. We use the term in order to designate those illnesses not generally defined and recognized in biomedicine.

Understanding local perspectives of the treatment of major children’s illnesses identified by the WHO [15], such as malaria [13], [16] and diarrhea [17], [18], as well as the treatment of children’s folk illnesses [19], [20], is essential to having a comprehensive understanding of childcare in Africa. In this study, we assessed how mothers make healthcare decisions by identifying which infant illnesses mothers in Western Africa treat with medicinal plants and for which illnesses they seek biomedical care or consult traditional healers. We worked in Bénin and Gabon, two African countries with diverse populations, vegetation types, cultures, and levels of human development. Our research was based on the following research questions: *Which children’s illnesses do Beninese and Gabonese mothers treat with medicinal plants? What are the major children’s folk illnesses in each country? For which ailments do mothers seek treatment from biomedical doctors? Which illnesses do mothers prefer to be treated by traditional healers?*

## Methods

### Study areas

Bénin is located in West Africa, with a surface area of 112,622 sq. km and a population of 9.8 million people [21]. It is ranked below the Sub-Saharan average in the Human Development Index (HDI) and considered a country of “low human development” [22]. It has an infant mortality ratio of 58 deaths per 1,000 live births [21]. Gabon is located in Western Central Africa, with a surface area of 267,667 sq. km, and a population of 1.7 million people [23]. The UNDP ranked Gabon 106th in the Human Development Index, slightly above countries of “medium human development” [24]. It has an infant mortality ratio of 48 deaths per 1,000 live births [23].

### Data collection and analysis

**Figure 1:**
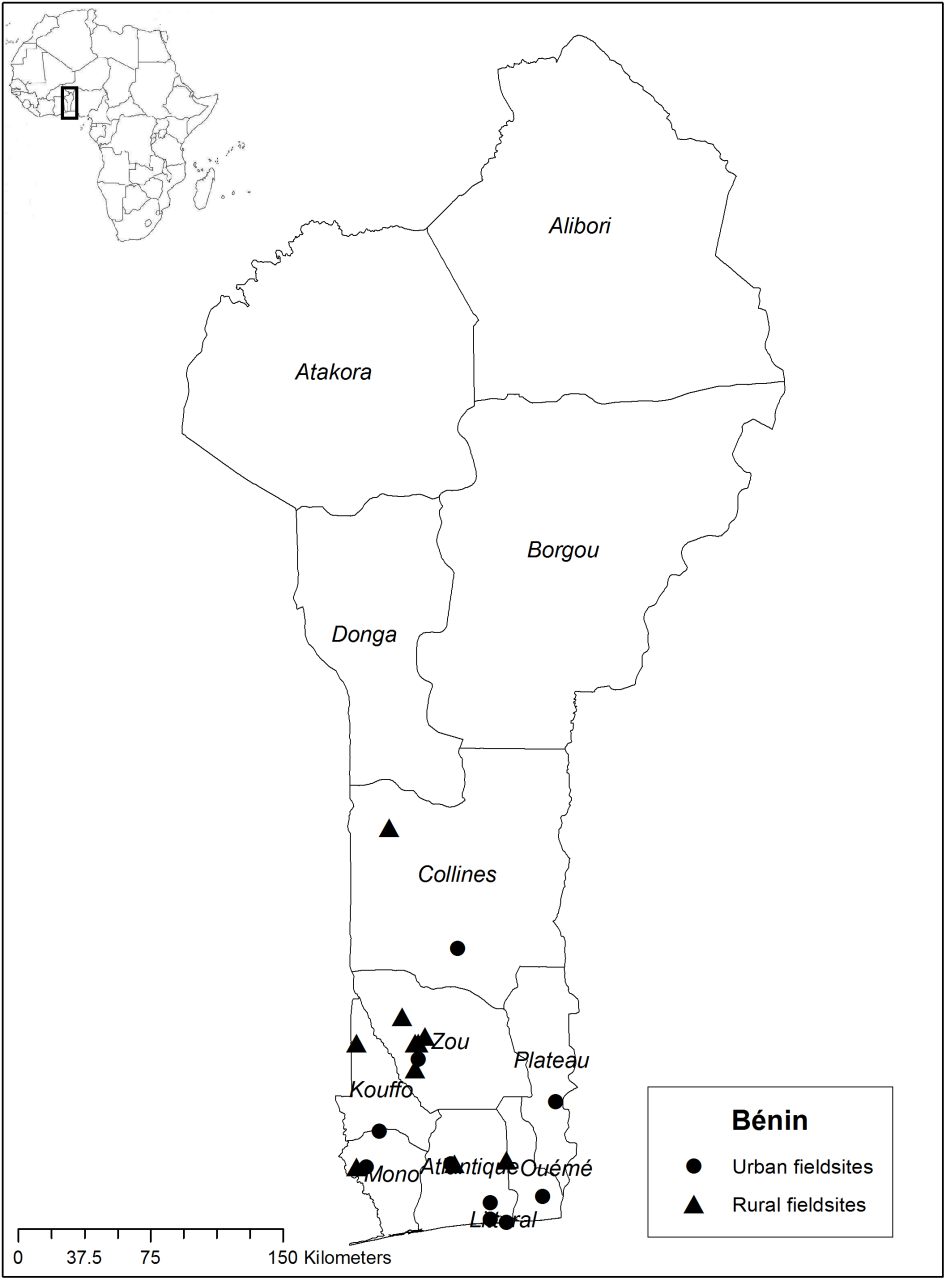
Map of the Bénin fieldwork sites in 2011.

**Figure 2:**
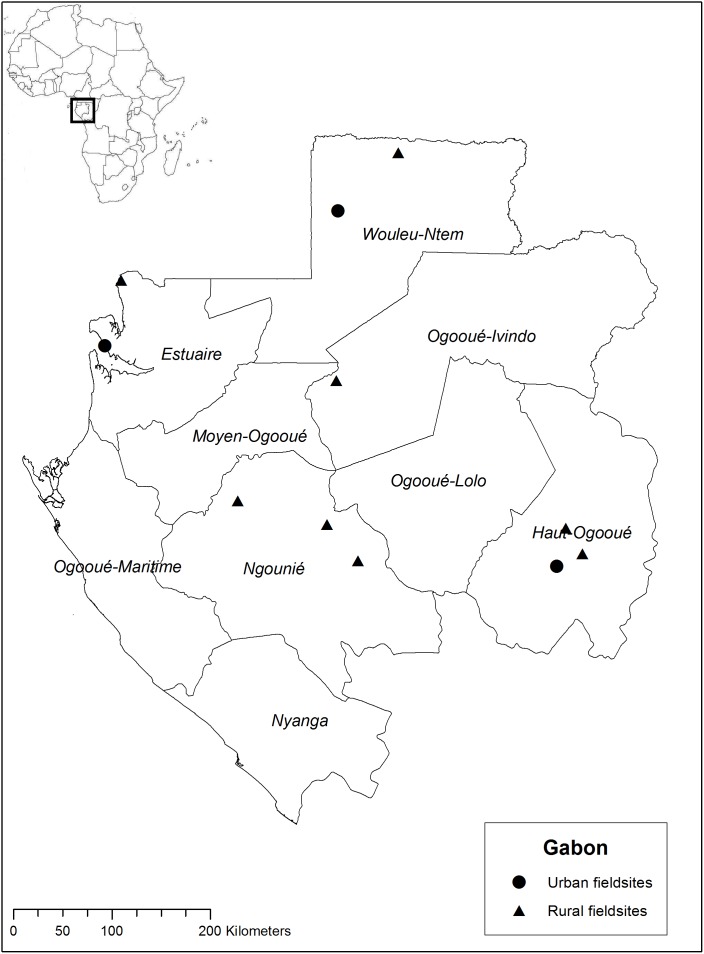
Map of the Gabon fieldwork sites in 2012.

Between April and October 2011 we worked in rural and urban areas of Bénin, mainly with Fon and Yoruba ethnic groups in the southern departments Collines, Kouffo, Zou, Plateau, Ouémè, Atlantique, Mono, and Littoral (Figure 1). From June until December 2012, we worked with Bantu-speaking ethnic groups in Gabon, namely, the Fang, Mitsogo, Obamba, and Bapounou peoples, in the departments of Estuaire, Woleu-Ntem, Haut-Ogooué, Ngounié, and Ogooué-Ivindo (Figure 2).

We started our research at the herbal medicine marketplaces in each country, taking time to familiarize ourselves with commonly utilized species, local illnesses and healthcare practices. From these initial market contacts, we utilized snowball sampling to identify women from surrounding urban and rural communities. We conducted an ethnobotanical questionnaire on practices related to childcare, including questions on herbal remedies for specific illnesses, definitions of folk illnesses, and preferences for the three types of healthcare. In total we interviewed 43 Beninese and 38 Gabonese mothers. In Bénin we worked with the following ethnic groups: Fon and related (70%), Yoruba and related (14%), Adja and related (5%) and mixed ethnicities (11%). In Gabon we worked with the following ethnic groups: Fang (45%), Mitsogo (16%), Babungu (16%), Obamba (8%), Bapounou (5%), and other (Ossimba, Omiene, Bateke) (10%). All women received financial compensation equivalent to local salaries for their time and involvement. We conducted the questionnaires orally in French, at participants’ homes and workplaces, and employed local language interpreters when needed.

After each of the 81 questionnaires, we accompanied participants to collect the plants that were cited in the interviews. We used standard botanical collection methods [25] to make vouchers of plants from the surrounding gardens, forests, and savanna landscapes. For women that we interviewed on the market, we purchased plants directly from market stalls and made trips into the field together to collect fresh samples when possible. In addition to collecting voucher specimens, we also gathered detailed information on their use, effects, and local names (see Tables S1 and S2). We deposited vouchers of all collected plants at the Herbier National du Bénin (BEN) and the Herbier National du Gabon (LBV). A complete set of duplicates was exported to the Wageningen branch of National Herbarium of the Netherlands (WAG), now merged with Naturalis Biodiversity Center (L), where the specimens were identified by the research team and several botanical specialists. Our plant collection did not involve endangered or protected plant species.

We assessed mothers’ knowledge of domestic medicine by calculating the number of species for each health issue and the percentage of mothers who knew at least one herbal recipe for each illness. We then summarized descriptions of folk illnesses and selected qualitative data from our interviews to illustrate which illnesses mothers treated with the three systems of healthcare: biomedicine, their own plant knowledge, or traditional healers. Maps of the fieldwork locations were created in ArcGIS 10.1 using open source geospatial data from DIVA-GIS (http://www.diva-gis.org/).

### Ethics statement

We adhered to all components of the Code of Ethics of the International Society of Ethnobiology [26], including carefully explaining the nature of our research, receiving oral consent, providing monetary compensation for involvement in the work, anonymizing informants' identities during data analysis, and working in a fully mindful and respectful manner. Oral consent was acquired in place of written consent due to the largely illiterate populations with whom we worked. We followed all research procedures and protocols at Leiden University, Naturalis Biodiversity Center, and the host institutes in each country. For the Bénin fieldwork, we acquired a formal invitation from the Faculté des Sciences Agronomiques, Université d’Abomey-Calavi (UAC), received a research permit (#041511) from the Faculté des Sciences et Techniques (UAC), and obtained a plant export permit (#0000591) from the Service de la Protection des Vegetaux et du Control Phytosanitaire, Ministre de l’Agriculture, de l’Elevage et de la Peche. For the Gabon fieldwork, the Centre National de la Recherche Scientifique et Technologique (CENAREST) provided a letter of invitation (#176). After approving our research proposal, CENAREST granted a research permit (#AR0028/12). We acquired authorization to enter the National Parks of Gabon (#000026) from the Agence Nationale des Parcs Nationaux (ANPN), and authorization to export botanical specimens (#00145, #00219) from the L’Institut de Pharmacopée et de Médecine Traditionelles (IPHAMETRA). We received formal administrative approval from our host institutes and were not required to submit our proposals to a human subjects review board for further review.

## Results

### Mothers’ knowledge of treating biomedical childhood illnesses with plants

Beninese participants cited 255 medicinal plant species and Gabonese participants cited 179 species. All species, together with vernacular names, scientific names and specific uses, are listed in Table S1 (Bénin) and Table S2 (Gabon). The highest percentages of plants in both countries were used to treat those child illnesses considered to be of major concern by the WHO: diarrhea, respiratory conditions, and malaria. Over 95% of participants from Bénin and over 84% of participants from Gabon knew at least one recipe to treat those diseases (Table 1). Respiratory-related ailments included illnesses such as the flu, cough, asthma, bronchitis, and specific folk illnesses related to respiratory problems in the case of Gabonese informants. Mothers also mentioned children’s ailments such as earache, chicken pox, colic, stomachache and vomiting, which we left out of the table because few plants and treatments were cited.

**Table 1:** Children’s health issues treated with medicinal plants by mothers in Bénin and Gabon.

Health Issue	# species(%) N = 255	# participants^1^(%) N = 43	# species(%) N = 179	# participants^1^(%) N = 38
	Bénin	Bénin	Gabon	Gabon
respiratory-related	53 (21)	42 (98)	49 (27)	32 (84)
diarrhea	39 (15)	41 (95)	27 (15)	34 (89)
malaria	54 (21)	41 (95)	36 (20)	33 (87)
intestinal cleanse*	58 (23)	41 (95)	31 (17)	33 (87)
measles	34 (13)	37 (86)	17 (9)	30 (79)
strengthener*	59 (23)	40 (93)	21 (12)	22 (58)
*fontanels* *	31 (12)	35 (81)	23 (13)	28 (74)
post-circumcision	32 (13)	37 (86)	14 (8)	21 (55)
*walk early* *	22 (8)	28 (65)	17 (9)	29 (76)
umbilical cord	13 (5)	32 (74)	12 (7)	24 (63)
convulsions/crisis*	32 (13)	33 (77)	4 (2)	4 (10)
teething	25 (10)	30 (70)	2 (1)	4 (10)
anti-sorcery*	21 (8)	25 (58)	6 (3)	6 (16)
fever	37 (15)	19 (44)	14 (7)	7 (18)
*atita* *	31 (12)	35 (81)	-	-
*ka* *	26 (10)	29 (67)	-	-
*fesses rouges* *	-	-	26 (15)	28 (74)
*la rate* *	-	-	34 (19)	26 (68)
*pogha* *	-	-	10 (6)	6 (16)

1 Percentage of mothers from each country who knew at least one herbal recipe.

*Folk illness or treatment.

### Children’s folk illnesses in Bénin

Mothers from Bénin mentioned two main folk illnesses, *atita* (Fon) and *ka* (Fon), and several cultural practices. *Atita* was described as a rash with “red bumps coming from the anus” or “itchy and stinging” red bumps in the groin and armpits. It was reported to be caused by the over-consumption of sugar or peanuts by the child or by the mother during pregnancy. The most common treatment for *atita* was an herbal bath or boiled plants consumed as tea (Table S1). *Ka* was described as an infection with large red bumps that were caused by the heat. It was reported to be treated by herbal baths, ingested teas, and through applying macerated plants directly to the infection.

The care and maintenance of open *fontanels* was a common practice in Bénin. Mothers’ considered it to be important for the soft spot of the fontanel to be able to “breathe” and eventually close. They used various herbal pomades, washes, and ingested teas for young children whom became ill from the failure of the fontanels to close. Beninese mothers highly valued their children to *walk early* in life. They encouraged their children with massages, herbal baths, and ingested teas. *Walking early* was seen as a sign that the child was developing normally and gaining independence, which would enable the mother to rest. Enemas were administered to newborn infants to remove the meconium, as well as to older infants for daily cleanses and constipation relief. These enemas frequently contained ground red peppers (*Capsicum annuum*) or different species of melegueta pepper (*Aframomum* spp.) mixed with water. Strengtheners were used in herbal treatments for premature birth, to strengthen newborns, and to assist in infant growth. Delayed and stunted growth were explained by mothers to be caused either by malnutrition or if an expectant mother came in contact with a praying mantis (hence the hunched over appearance and thin arms of an infant). It was treated with an herbal bath with an herbal recipe that included the eggs of a praying mantis. Various herbal treatments were applied to the umbilical cord of newborns to hasten the recovery period, as well as the application of herbs to assist in the healing process of circumcision.

### Children’s folk illnesses in Gabon

Gabonese and Beninese mothers shared the cultural practices of monitoring the closure of the *fontanels*, encouraging children to *walk early*, and bathing newborns and young children to give strength. Monitoring the closure of *fontanels* (*abobane* in Fang) in Gabon was considered necessary to avoid “bad wind or spirits” that could enter, resulting in a child’s stunted growth. Herbal treatments included applying pomade made from leaves directly to the infants’ head and applying peanut butter to the palate of the mouth (Table S2). Mothers pointed out that not all children suffered from open *fontanels*. Encouraging children to *walk early* also was seen as the mothers’ recuperation of independence; they could do more work because the child could play outside with its siblings. One of the most commonly mentioned Gabonese folk illnesses was known as *fesses rouges* in French (*ntcheke* in the Babungu language, *kusu* in Punu, *tzogho* in Fang, and *kengey* in Teke). Like its literal French translation, the main symptom of *fesses rouges* was a red, irritated bottom caused “by sitting in the dirt,” “by microbes,” or “during childbirth when heat enters the body through the anus”. Treatments included applying herbal pomades and herbal enemas.

Folk illness *la rate (tzit* in Fang and *kabama* in Teke*)* which in English is translated as “the spleen,” was characterized by a tender, swollen left side of the body and a skinny overall physical build. An earlier stage of *la rate*, known as *ebem* in Fang, was characterized by high fever and green feces. Although most respondents were not aware of the cause of *la rate*, some participants mentioned God’s will, anemia, and malnutrition as possible causes. Treatments included herbal massages, herbal enemas, and traditional “vaccinations”- the creation of small incisions on the left side of the body with a razor blade and application of the fresh juice of plants into the cuts. Folk illness *pogha* (in *Mitsogo* and *Babungu* languages) was characterized by fever, fatigue, convulsions, but distinct from the symptoms of malaria. It was reported to be caused either by God’s will or the mother’s food consumption when the child was young. Herbal baths were the primary form of treatment. Included in the calculations for respiratory-related ailments (Table 1) were several recipes mentioned by Fang women for respiratory-related folk illnesses, including *onkoe abijel*: “respiratory problems caused by bad water during delivery,” *onkouabial*: “bad lungs after birth,” and *ebulonkuk*: “bad lungs caused by sorcery”.

### Mothers’ knowledge of treating folk illnesses with plants

Aside from the use of plants for intestinal cleansing, fewer women knew how to treat folk illnesses than biomedical illnesses (Table 1). In Bénin, percentages of mothers who knew recipes for them ranged from 80% for *atita* to 65% for *ka*. In Gabon, over two-third of all participants knew herbal treatments for common children’s folk illnesses. With the exception of *fontanels* and *walk early*, Beninese folk illnesses like *atita* and *ka* were unknown to Gabonese mothers, while *fesses rouges* and *la rate* were not known in Bénin. Although the terms and perceived causes of *atita* in Bénin and *fesses rouges* in Gabon do not coincide, the two folk illnesses were somewhat similar in description. The folk illness *pogha* was only mentioned as an illness by mothers in the Gabonese department of Ngounie.

### Health-seeking behaviors of Beninese mothers

Although there was little consensus on one preference for healthcare (Table 2), Beninese women in our study generally reported starting to treat their children with medicinal herbs, following up with biomedical care, and seeking traditional healers as a third resort. An 80-year old Mina woman said *“*Traditional medicine is first. Some use the hospital first, for example for fever or if one needs blood. A traditional healer is called upon to consult the *fa* (oracle) and for sacrifices”. Women who reported to never consult traditional healers mentioned the church and prayer as spiritual forms of treatment. Self-administered herbal medicine was reported to be preferred for treating children’s illnesses due to its ability to help defecate well, its use as preventative medicine, and its perceived effectiveness. Respondents often mentioned using plants to self-treat for a certain number of days (ranging from two days to one week) and then seeking biomedical care. Biomedicine was acknowledged to have the advantage of having advanced technology and materials but was perceived as being more expensive. A 36-year old Yoruba woman said, “Traditional medicine is used for constipation and *atita*- those you can treat at home. Modern medicine is used for difficult cases- they are better equipped. Traditional healers are consulted for superhuman cases because they know more about this domain”. Advanced forms of illnesses, especially malaria, were commonly reported to be treated with biomedicine. Seeking traditional healers to treat victims of sorcery and folk illnesses were strong themes. Traditional healers were reported to treat illnesses “that surpass the knowledge of doctors,” and for causes such as sorcery or witchcraft. A minority of mothers reported that common folk illnesses and asthma were “men’s knowledge,” outside of the maternal domain of skills. It was not clear if men’s knowledge meant the specialized knowledge of (male) traditional healers or more generally, fathers in the community. An 80 year old Fon woman said, “First try to treat at home with herbs for a couple of days. If they do not work, go to the hospital. If this does not work, go to a traditional healer. Asthma and fetus health are men’s knowledge. *Fontanels* are traditional healers’ knowledge”.

**Table 2:** Most frequent responses by mothers to healthcare seeking options question in Bénin (N = 43) and Gabon (N = 38).

Response	% of mothersBénin	% of mothersGabon
**Ranking of three health care options**		
First choice self-treatmentwith plants	42	29
First choice biomedicine(malaria, anemia, fever)	16	32
First choicebiomedicine (always)	0	21
First choicetraditional healer	7	18
Second choicebiomedicine	30	13
Second choice self-treatmentwith plants	0	11
Third choicetraditional healer	23	3
Never consulttraditional healer	5	11
**Healthcare choice for specific cases**		
Traditional healerfor sorcery	44	5
Biomedicine for advancedcases (malaria, anemia)	35	5
Self-treat with plantsfor specific illnesses(diabetes, measles, stomachache)	21	13
Self-treat with plantsfor simple cases(malaria, diarrhea)	28	0
Traditional healerfor specific cases(fontanels, paralysis)	12	8
Men for specificillnesses (walk early, asthma)	9	0

### Health-seeking behaviors of Gabonese mothers

There was also a large range of responses from the Gabonese women involved in our study (Table 2). Nearly the same number of Gabonese mothers preferred self-treatment as a first form of healthcare as mothers who preferred treating children first with biomedicine. The strongest consensus of women cited specific illnesses, especially malaria, in which they would seek biomedical care directly. A 40-year old Obamba woman said “Use modern medicine for malaria, etc. We’re evolved for serious illness. Use traditional medicine if modern medicine doesn’t work, or if it’s not serious. A *ganga* is outdated, we no longer use them”. However, other women favored the consultation of a *ganga,* the spiritual leader of the community, or the *nyembe*, the spirit in a women’s secret society, in order to know where to treat the illness. This was a reoccurring theme, suggesting a strong the role of spirituality and religion in childcare, especially for folk illnesses. A 50 year old Fang woman said, “One should seek modern medicine for an operation; injections go straight to the blood and therefore work faster… Traditional medicine depends on God’s grace; prayer helps too. Go to a *ganga* for sorcery”. We found a reoccurring theme among Gabonese mothers that three systems were largely complementary. A 42-year old Fang woman said: “Try traditional medicine, if it does not work, the *genies* (spirits) will tell you to go to modern medicine. Work with the spirits! Between modern medicine and traditional medicine, there is a good collaboration. Gabon is currently in good position between the two systems”. A 61-year old Omiene women said “The three systems are complementary; you will find a solution between the three. It also depends on one’s belief system; some people are hesitant to go to a *ganga*”.

## Discussion and Conclusion

### Biomedical illnesses and their treatment

The majority of participants from Bénin and Gabon knew herbal treatments to treat the top causes of infant mortality: respiratory problems (98%, 84% respectively), malaria (95%, 87%), and diarrhea (95%, 89%). This outcome suggests that traditional medicine, and more specifically mothers’ knowledge of plants, is a major factor in the management of these common childhood health ailments. Even though mothers were knowledgeable on treating these illnesses, however, they also distinguished situations where they would seek biomedical care prior to using domestic medicine, such as complicated cases of malaria, anemia, or fever. Studies in other African countries also found that mothers preferred to treat malaria with biomedical care [27]. Only a few mothers mentioned diarrhea specifically as a case that they would seek biomedical care as a first option, suggesting diarrhea is largely treated by mothers with plants as was found in a recent study in Sierra Leone [28]. Likewise, respiratory ailments were not specifically mentioned as a case for seeking biomedical care. The high percentage of women who know how to treat these illnesses and the high number of plants attributed to their treatment suggest a parallel recognition of major causes of infant morbidity and mortality between the mothers and the statistics of the WHO. This agreement between medical priorities is not always the case, in a similar study on women’s health in Bénin and Gabon, we found that local and biomedical priorities did not coincide [29].

### Folk illnesses and their treatment

Folk illnesses ranked directly after the major biomedical illnesses for children in terms of mothers’ medicinal plant knowledge. Our research supports ethnobotanical studies from other parts of the world that have indicated local populations commonly prefer to treat folk illnesses with traditional medicine [5], [30]–[31]. While many participants in our study knew herbal remedies to treat folk illnesses, it is clear that traditional healers and religion have a strong role in this domain. Men, more generally speaking, were also regarded as having specialized knowledge in Bénin. Fathers also have a role in the treatment of children’s illnesses, in terms of their own knowledge of medicinal plants [6] and their role in family decision-making [27].

Folk illnesses are of interest to biomedical health care providers, not only because they often make up a significant portion of local health complaints [5] but they may address underlying neglected diseases. Fontanels are common children’s folk illnesses around the world, and in other African countries such as Swaziland, Zimbabwe, Botswana and Malawi [32]. Certain (bulging or sinking) appearances of the fontanels may be symptoms of a range of disorders from dehydration to malnutrition to Down Syndrome [33]. Moreover, when mothers apply paste on the fontanel prior to arriving at the hospital, doctors cannot fully assess the fontanel (because of the plant pomades) and may misdiagnose the child’s illness. *La rate* resembles the symptoms of sickle-cell disease, a common yet neglected illness of children in Western Africa [34], especially its characteristic concentrated pain on the left side and spleen enlargement [35]. This overlap is a fertile ground for improved research and educational programs on sickle cell disease [36]. Enemas for intestinal cleanses, especially for newborns and small children, were a common practice in both countries. In the Ivory Coast, Gottlieb [37] found that enemas were used to make a baby defecate at a given time. Biomedical research has highlighted the danger in using enemas, especially among young children [38].

Even if these illnesses are not recognized as biological in nature, their treatment nevertheless has consequences, either positive or negative, on children’s health. Taking local perspectives and treatments into account not only informs biomedicine of cultural concepts of illness and healing [39], it also facilitates an understand of plant’ effects through pharmacological studies [40], and enables an understanding of how traditional systems of healing and biomedicine are already interacting on the ground [41].

### Complementarity of three systems

The lack of any one definitive pattern of healthcare-seeking behavior among mothers in our study reflected the truly pluralistic healthcare systems of both countries [1], the dynamic process of deciding how to care for children [15], and the fact that mothers see the three African systems of healthcare as largely complementary. Mothers’ general *pattern of resort* [42] was to self-treat with plants first, seek biomedical care for specific illnesses or as a second source of healthcare and to consult the spiritual realm, including *ganga*s and the *nyembe* in Gabon, to treat folk illnesses. However, as found in a recent study in South Africa [43], this pattern varied according to illness; each healthcare option was seen to have specific advantages and disadvantages. Biomedicine was perceived to have the advantage of advanced technology and materials, especially for treatments related to blood transfusions. Some mothers in Bénin reported a preference of using self-collected herbal medicine over biomedical care due to the expensive of modern treatment.

Future research can take demographic and socio-economic data into account to further the understanding of preferences for childcare treatment [28]. Additionally, our research did not explore mothers’ attitudes towards the quality of the different healthcare options. Future studies can ask women if a positive experience with one form of treatment would influence future decisions. Infant and child healthcare will be enriched if local knowledge, illness concepts, and medicinal plants fit into a larger framework that studies healthcare from a community perspective [1], including researchers from outside the biomedical field [5]. With the Millennium Development Goals concluding in 2015, and the reality that both countries have not met their targets of reducing infant mortality rates [22], [24], there is a renewed opportunity for infant healthcare initiatives to become more comprehensive.

## Supporting Information

Table S1 **Species cited in 43 questionnaires in Bénin: scientific botanical name, vernacular plant name(s), plant part used, preparation, use category and collection number.**
(DOCX)

Table S2 **Species cited in 38 questionnaires in Gabon: scientific botanical name, vernacular plant name(s), plant part used, preparation, use category and collection number.**
(DOCX)
