# Supplementary material for: Traditional Medicine and Childcare in Western Africa: Mothers’ Knowledge, Folk Illnesses, and Patterns of Healthcare-Seeking Behavior
Source: PLoS One. 2014 Aug 22;9(8):e105972. doi: 10.1371/journal.pone.0105972 (PMC4141852; doi:10.1371/journal.pone.0105972)
Supplement: Table S1 — Species cited in 43 questionnaires in Bénin: scientific botanical name, vernacular plant name(s), plant part used, preparation, use category and collection number. (DOCX) [file pone.0105972.s001.docx]

**Table S1**

Species cited in 43 questionnaires in Bénin: scientific botanical name, vernacular plant name(s), plant part used, preparation, use category and collection number

| Botanical Name | Vernacular Name^a^ | Used part | Preparation^b^ | Use category^c^ | AMT #^d^ |
| --- | --- | --- | --- | --- | --- |
| *Abelmoschus esculentus* (L.) Moench | gombo (Fr) | leaves, fruit | HB, EA | newborn, fontanels | NC |
| *Abrus precatorius* L | bouchenchen (T), djindjeklendjeman/viviman (F, G) | leaves | E, T, HB | cough, post-circumcision, respiratory problems | 297 |
| *Acacia* cf. *erythrocalyx* Brenan | ewan (N) | stem | HB | walk early | NC |
| *Acacia* cf. *sieberiana* DC. | adouwe (G) | leaves | T | teething | NC |
| *Acacia nilotica* (L.) Delile | banni (F) | seeds | T | asthma | NC |
| *Acanthospermum hispidum* DC. | toba/ahowonglon (F), kpononnou (G), owgboman (N), tchako (T) | leaves, whole plant | D, T, EN | cough, malaria, fever, CBD ka, respiratory problems, walk early | 211, 237 |
| *Acrostichum aureum* L. | sofofo (G) | leaves | T | walk early | 428 |
| *Adansonia digitata* L. | kpassa (F), baobab (Fr), | leaves, bark | HB | premature birth, strengthener | NC |
| *Adenia cissampeloides* (Planch. ex Hook.) Harms | akolebodjou (N) | leaves | T | malaria | 445 |
| *Aframomum melegueta* K.Schum. | atakounkui (Y,N) | fruit | HB | measles | NC |
| *Agelaea pentagyna* (Lam.) Baill. | ahwahazoun (F,G) | leaves | T | strengthener, stomach ache, intestinal cleanse | NC |
| *Ageratum conyzoides (*L.) L. | suyonou (G), legboku (K) | whole plant | T | respiratory problems, fever | 430, 530 |
| *Albizia* cf. *adianthifolia* (Schum.) W.Wight | ayolo (F) | wood | T | asthma | NC |
| *Alchornea cordifolia* (Schumach. & Thonn.) Müll.Arg. |  | leaves | T | CBD atita, convulsions | 631 |
| *Allium sativum* L | l'ail (Fr), aiyo (F,G,N) | stem | D, T, A, HB, EN | convulsions, constipation, intestinal cleanse, measles, CBD atita, diarrhea, fontanels | NC |
| *Allium* sp. | ayomanwoniwono (F) |  | T | constipation | NC |
| *Aloe macrocarpa* Tod. | aloes (Fr) | exudate | D | constipation | NC |
| *Alternanthera pungens* Kunth | inchako (T), baglon (A) | leaves | EN, T | walk early, anti-sorcery, malaria | 236, 239, 490 |
| *Amaranthus viridis* L. | amadjin (F) | leaves, whole plant | EA | CBD ka, measles | 582 |
| *Ampelocissus leonensis* (Hook.f.) Planch. | adoyo/teple (F), ecama (A) | whole plant | T | cough, malaria | 408, 463 |
| AMT 141 | weydumey (M) | whole plant | HB | anti-sorcery | 141 |
| AMT 265 | kanchino (T) | bark | T | diarrhea | 265 |
| *Pleurotus tuber-regium*  *(Rumph. ex Fr.) Singer 1951* | aisankoum (F) | fungus | E | asthma | 601 |
| *Anacardium occidentale* L. | kanghougoto (F,G), canjew (T) | bark | T | cough, respiratory problems, asthma, teething, post-circumcision | 425,257 |
| *Ananas comosus* (L.) Merr. | ananas (Fr) | fruit | T | malaria | NC |
| *Annona muricata* L. | shapshap (M) | leaves | D | asthma | 134 |
| *Anthocleista vogelii* Planch. | gontoudo (F), goussouedo (G), irakpo (T) | root, wood | T | intestinal cleanse, stomachache, meconium removal, constipation | 281 |
| *Arachis hypogaea* L. | arachide (Fr) | leaves |  | fetus strengthener | NC |
| Arecaceae sp. | gueyo (F,G) | leaves | T | walk early | 427 |
| Arecaceae sp. | itowonti (A) | seeds |  | anti-sorcery | 477 |
| *Argemone mexicana* L. | ahondja/wetcheyon (F, G, N, Y), magele (T) | leaves | T, HB, EA | newborn strength, malaria, meconium removal, stomachache, intestinal cleanse, fontanels, fever | 233, 492, 609 |
| Asteraceae sp. | atanatebe (T) | leaves | E | constipation | 292 |
| *Azadirachta indica* A.Juss. | neemma/kininma (F), lili (T) | leaves | T, HB | stomach ache, vomiting, malaria, measles, convulsions | 274 |
| *Baphia nitida* Lodd. | susupeyma (F) | leaves, wood | HB, EA | newborn strength, CBD ka | NC |
| *Barteria* cf. *nigritana* Hook.f. | okoukou (F,N), okotcho (Y) | bark, leaves | HB | newborn strength, strengthener, premature birth | 451 |
| *Bauhinia thonningii* Schum. | kloma (F), akluema (A) | leaves | EA, T | toothache, strengthener | 466, 560 |
| *Blighia* cf. *sapida* K.D.Koenig | lissekui (F) | seed | E | asthma | NC |
| *Blighia* cf. *unijugata* Baker | agbovian (F, G) | bark | T | diarrhea | NC |
| *Boerhavia diffusa* L. | kasualee (F) | leaves | HB | CBD ka | 467 |
| *Bombax* cf. *buonopozense* P.Beauv. | aloviaton (F) | exudate, leaves | D, EA | cough, post-circumcision | NC |
| *Bridelia ferruginea* Benth. | honssounkuékué (F) | leaves, bark, root | HB, T | CBD atita, strengthener, asthma, walk early, newborn strength, CBD ka, convulsions, post-circumcision | NC |
| *Bryophyllum* cf. *pinnatum* (Lam.) Oken | afoman (N), affiman (G) | leaves | T | walk early | NC |
| *Caesalpinia bonduc* (L.) Roxb. | agekwin (A,F,G) | seeds, leaves | AT, T | anti-sorcery, CBD atita, newborn strength, intestinal cleanse, constipation | 517 |
| *Caesalpinia pulcherrima* (L.) Sw. | tegbesu (F), orgueil de chine (Fr) | leaves | HB, T | CBD atita, preventative, convulsions, asthma | NC |
| *Cajanus cajan* (L.) Millsp. | kulikwun, klema (F), pulema (K), kolo (N, T) | leaves | T, HB | measles | 255, 497, 551 |
| *Calotropis gigantea* (L.) Dryand. | wagashima (A, F, K, M), pbento (F) | leaves | T, D, EA | anti-sorcery, cough, umbilical cord, measles, strengthener, asthma | 469 |
| *Calotropis procera* (Aiton) Dryand. | bambamo (T) | leaves | EA | umbilical cord | 285 |
| *Capsicum annuum* L. | piment (Fr), vavofli-fliman (G) | fruit, whole plant | EN, EA, T | toothache, intestinal cleanse, wounds, convulsions | 439 |
| *Carica papaya* L. | pbema (F), Kpinman (N, Y) | seeds, leaves | T, HB, T, D | malaria, strengthen, constipation, fever | NC |
| *Carissa spinarum* L. | aheyhey (F) | whole plant | EA | walk early | NC |
| *Cassia sieberiana* DC. | agbilikopao (T) | wood | T | vermifuge | 280 |
| *Cassytha filiformis* L. | agbegbekan (F, G) | whole plant | T, HB | fontanels, strengthener | NC |
| *Ceratotheca* cf. *sesamoides* Endl. | agboma (F) | leaves | EA | fontanels | NC |
| *Chamaecrista mimosoides* (L.) Greene | kinafimitche (F,G,N) | whole plant | T | anti-sorcery | NC |
| *Chamaecrista rotundifolia* (Pers.) Greene | azima (F) | leaves, whole plant | T | malaria, constipation, newborn strength | NC |
| *Chassalia kolly* (Schumach.) Hepper | atindjedo (G), okpao (Y), akpa (N) | root, leaves | T, EA, HB | intestinal cleanse, fontanels, post-circumcision | NC |
| *Chromolaena odorata* (L.) R.M.King & H.Rob. | agatou (F, N, T), gueflu (K) | leaves | EA, HB | post-circumcision, fever, headache | 251, 448, 499 |
| *Citrullus colocynthis* (L.) Schrad. | kakanya (T) | leaves | EA | vermifuge | NC |
| *Citrullus lanatus* (Thunb.) Matsum. & Nakai | goussi (F,G) | fruit | T | intestinal cleanse, constipation | NC |
| *Citrus aurantiifolia* (Christm.) Swingle | cleman (F,G), citron (Fr) | leaves, fruit, root, bark | T, D, E, A, HB | constipation, intestinal cleanse, measles, convulsions, malaria, stomachache, cough, meconium removal, clear throat of newborn, respiratory problems, vomiting | 264 |
| *Citrus* sp. | orange (Fr) | skin from fruit | EA | wounds | NC |
| *Clausena anisata* (Willd.) Hook.f. ex Benth. | gbozoun (F), gbossouazowin (G), arukoumbo (T) | leaves | HB, T, E | CBD atita, constipation, newborn strength, umbilical cord, headache, cough | 260, 426, 442, 454 |
| *Cleistopholis patens* (Benth.) Engl. & Diels | honsoungoto (F), housinkoman (G) | bark, leaves | T | constipation, stomachache, teething | NC |
| *Cleome gynandra* L. | khaya (M) | leaves | D | yellow fever, earache | 139, 486 |
| *Cleome viscosa* L. | akaya (F), kayasu (M) | leaves | M, HB | teething, walk early | 136,604 |
| *Clerodendrum* cf. *capitatum* (Willd.) Schumach. & Thonn. | weman/wedo (F,G) | leaves, root | T, HB | CBD ka, CBD atita, malaria, fever | NC |
| *Cnestis ferruginea* Vahl ex DC. | gbovian | leaves | D, T, HB | diarrhea, measles | 209 |
| *Cocos nucifera* L. | agodo (F, G) | root | T | malaria, constipation, intestinal cleanse | 370, 461 |
| *Cola millenii* K.Schum. | aloviaton (A) | leaves | T | malaria, fever | NC |
| *Combretum* cf. *grandiflorum* G.Don | adoucito (F, G, N) | leaves | T | diarrhea, teething | NC |
| *Combretum collinum* Fresen. | bodumey (T) | root | D | CBD atita | 294 |
| *Combretum micranthum* G.Don | kinikiniba (F, G, N, Y) | leaves | EA, D, HB, T | measles | NC |
| *Combretum* sp. | adouco (F), adoukin (G) | leaves | T, HB | measles, diarrhea, teething, fontanels, anti-sorcery | 400, 405 |
| *Commiphora africana* (A.Rich.) Endl. | feliyimi (G), origi (T) | leaves, branch | T, AT | cough, convulsions | 434 |
| Convolvulaceae sp. | eweyeye (G) | whole plant | T | cough | 435 |
| *Corchorus olitorius* L. | crencren (F) | leaves | D | malaria, constipation | NC |
| *Costus afer* Ker Gawl. | teteglete (F) | leaves | E | protection against accidents | 636 |
| *Crateva adansonii* DC. | hontonzouzouin (F, G) | leaves, root | HB, T | post-circumcision, CBD ka, anti-sorcery, intestinal cleanse, malaria | 135, 613 |
| *Crescentia cujete* L. | treci (A), ka (F), calebasse (Fr) | fruit, leaves | EA, T | convulsions, CBD ka | 489 |
| *Crotalaria* cf. *retusa* L. | awiyan (F) | leaves | EA | fontanels, post-circumcision | NC |
| *Croton gratissimus* Burch. | hèmandédji (F, G), adjekofole (N, Y) | leaves | T, EA, E, HB | anti-sorcery, measles, fever, CBD ka | 456 |
| *Cucumis metuliferus* E.Mey. ex Naudin | gboounon (F) | fruit | T, A | measles | NC |
| *Cyanthillium cinereum* (L.) H.Rob. | mayantin (F), houssinkussè (F, G) | leaves, whole plant | EA, D, HB, T | CBD atita, post-circumcision, walk early, premature birth | 410, 473 |
| *Cymbopogon* sp. | timan (F) | leaves | T | malaria | NC |
| *Cymbopogon citratus*(DC.) Stapf | citronelle (F, Fr) | leaves | T | intestinal cleanse, strengthener, meconium removal | NC |
| *Cynometra megalophylla* Harms | foladgoto (F), bougoto (G) | bark | HB, T | CBD atita, newborn strength | 453 |
| *Daniellia oliveri* (Rolfe) Hutch. & Dalziel | zanlinkpon (F), eweiya (N, Y), inya (T) | resin, bark, leaves | HB, T | anti-sorcery, walk early | 286, 462 |
| *Dennettia* cf. *tripetala* Baker f. | iberi (T) | fruit | EA | umbilical cord | NC |
| *Desmodium velutinum* (Willd.) DC. | trèdoavohou (F, G) | leaves | T | asthma, cough, diarrhea, fontanels, teething | 415, 468 |
| *Dialium guineense* Willd. | atituey (M) | leaves | T | malaria | 148 |
| *Dichapetalum madagascariense* Poir. | gbaglo (A, F, G) | leaves | T, HB | malaria, fever, convulsions, measles, CBD ka | NC |
| *Dichrostachys cinerea* (L.) Wight & Arn. | badawouin (F) | root | T | measles | NC |
| *Dracaena fragrans* (L.) Ker Gawl. | anyama (K) | leaves | EA | ear ache | 533 |
| *Dysphania ambrosioides* (L.) Mosyakin & Clemants | godo (F), azobidi (K) | whole plant, leaves | HB, EA, T | newborn strength, post-circumcision, asthma, fontanels, vermifuge | 557 |
| *Eclipta prostrata* (L.) L. | zoma (F) | leaves | HB, T | post-circumcision, malaria | 596 |
| *Ehretia cymosa* Thonn. | kanbala (F), miyonman (G) | leaves | T | diarrhea, malaria, fever | 460 |
| *Elaeis guineensis* Jacq. | tjotjo (F), huile rouge (Fr), inkiyo (T) | oil from seed | EA, T | wrinkly newborns, fontanels, convulsions, wounds, fever, measles, respiratory problems, umbilical cord | NC |
| *Entada gigas* (L.) Fawc. & Rendle | gbagbla (F) | seeds | T, HB | constipation, intestinal cleanse, constipation | 418 |
| *Erythrina* cf. *senegalensis* DC. | pbaklesi (F) | leaves | HB | diarrhea | 559 |
| *Erythrococca anomala* (Juss. ex Poir.) Prain |  | leaves |  | teething | 495 |
| *Eucalyptus* sp. | eucalyptus (Fr) | leaves | T, HB | cough, respiratory problems, malaria | NC |
| *Euphorbia hirta* L. | anossikan (G) | whole plant | HB | measles | NC |
| *Evolvulus* cf. *alsinoides* (L.) L. | droman (G) | leaves | T | teething | NC |
| *Indigofera* sp. | fonvi (F,G,N) | whole plant | T, HB | walk early | 429 |
| Fabaceae sp. | vonsou | seeds | E, EA | diarrhea, fontanels | NC |
| *Ficus* cf. *lutea* Vahl | adako (T) | bark | T | diarrhea | NC |
| *Ficus exasperata* Vahl | igpi (T) | sap | EA | umbilical cord | 252 |
| *Ficus sur* Forssk. | voma (F), oweyokpoto (F, G), okpoto (Y) | leaves, bark | HB, T | fever, strengthener, premature birth | 579 |
| *Flacourtia indica* (Burm. f.) Merr. | agbonkadjè (F) | leaves, root | T, HB | diarrhea, measles | NC |
| *Flueggea virosa* (Roxb. ex Willd.) Royle | tchèkè-tchèkè (F), ayiku (T) | leaves | T, HB, E, D | constipation, strengthener, meconium removal, convulsions, intestinal cleanse, teething, malaria | 276, 569 |
| *Garcinia kola* Heckel | ahowe (F) | seeds | T, HB | newborn strength, intestinal cleanse | NC |
| *Garcinia* sp. | ahowé/kola (F) | leaves, seeds | HB, T, E | newborn strength, malaria, anti-sorcery, fontanels, diarrhea | 419 |
| *Gardenia ternifolia* Schumach. & Thonn. | dakplasou (F) | leaves | D | malaria, fetus strengthener | NC |
| *Gladiolus dalenii* Van Geel | baka (F) | tuber | E | asthma | NC |
| *Glycine max* (L.) Merr. | soja (F) | seeds | D | constipation | NC |
| *Gmelina* cf. *arborea* Roxb. | fiofiotin (F) | leaves | T | constipation | NC |
| *Hackelochloa granularis* (L.) Kuntze | azosongo (F, G) | whole plant | T | strengthener | NC |
| *Heliotropium indicum* L. | kokolosutepadjay (F), koulodin (N) | whole plant | HB, T | fever, CBD atita, CBD ka | 447 |
| *Heterotis* cf. *rotundifolia* (Sm.) Jacq.-Fél. | hèhèman (F) | leaves | T | anti-sorcery, fever, convulsions, malaria, post-circumcision | NC |
| *Hibiscus acetosella* Welw. ex Hiern | hungbe (A), yangba (F) | leaves | T | strengthener, malaria | 465, 594 |
| *Hibiscus* sp. | podey (M) | leaves | T | malaria, fever | NC |
| *Hibiscus surattensis* L. | kpofin | whole plant | T | anti-sorcery | NC |
| *Hoslundia opposita* Vahl | klongble (G) | leaves | HB | strengthener | 437 |
| *Hygrophila auriculata* (Schumach.) Heine | hosugoto (K) | bark | T | asthma | 496 |
| *Hymenocardia acida* Tul. | fefeya (T) | leaves | E | teething | NC |
| *Hyptis suaveolens* (L.) Poit. | sonsupeypeyma/koueflou (F), kouloubi (T) | leaves | HB, T, E | fever, mosquito repellant, diarrhea, CBD atita, CBD ka, dysentery | 291, 406, 472 |
| *Icacina* cf. *trichantha* Oliv. | agebebema (F) | leaves | T | diarrhea | NC |
| *Imperata* cf. *cylindrica* (L.) Raeusch. | seman (F, G), eweekan (N) | leaves | T | teething, respiratory problems | NC |
| *Indigofera* sp. | ahoobey (A) | leaves | T | strengthener, constipation | 366 |
| *Jatropha* cf. *curcas* L. | babaki (A), ajakpotu (F), eweakporo (N, Y), kitipopo (T) | leaves, branch | T, HB, SB | malaria, fever, intestinal cleanse, convulsions | 659 |
| *Jatropha multifida* L. | wêkêman (F) | leaves | T | CBD atita | 403 |
| *Jatropha* sp. | jatrophado (N) | root | T | respiratory problems | NC |
| *Justicia flava* (Vahl) Vahl | tchoutchouglouchou (F, G) | whole plant | E, HB | anti-sorcery, newborn strength, fontanels | 633 |
| *Kalanchoe crenata* (Andrews) Haw. | afaman (F, Y), adodo (T) | leaves | EA, D | umbilical cord, cough | 261 |
| *Kedrostis foetidissima* (Jacq.) Cogn. | tchiyoman (F, G) | leaves | T | convulsions, malaria, fever | NC |
| *Khaya senegalensis* (Desv.) A.Juss. | zounsa (F, N), agao (T) | bark, leaves | HB, T, A, EA | newborn strength, walk early, teething, strengthener, convulsions, premature birth, malaria, CBD ka, CBD atita, anti-sorcery, post-circumcision | 244, 284 |
| *Kigelia africana* (Lam.) Benth. | ylanblikpo (F), pando (T) | bark | T, HB | constipation, intestinal cleanse | 249, 458, 577 |
| *Lagenaria breviflora* (Benth.) Roberty | obiri (N, Y) | whole plant, leaves | T, HB | CBD ka | 446 |
| *Lagenaria* cf. sp. | iytrue (A) | leaves | T | cough | NC |
| *Lannea acida* A.Rich. | zuzugoto (F), aku (T) | bark | HB, T | walk early, newborn strength, strength | 282 |
| *Lannea barteri* (Oliv.) Engl. | hounman (F) | leaves | T, HB | premature birth, strengthener | NC |
| *Lannea* sp. | mangbevide (F) | bark | T | convulsions | NC |
| *Lantana camara* L. | hlatchayo (F, G, N, Y) | leaves | T, HB | post-circumcision, diarrhea, CBD atita, CBD ka | 404 |
| *Lawsonia inermis* L. | laliman (F, G) | leaves | T | malaria | NC |
| *Lecaniodiscus* cf. *cupanioides* Planch. ex Benth. | ganotun (F, G) | leaves | HB | premature birth | NC |
| *Lippia multiflora* Moldenke | yonya (F, G), yeye/tchaga (T) | leaves | T, HB, EA | anti-sorcery, diarrhea, CBD atita, CBD ka, post-circumcision, teething, cough | 270, 402 |
| *Lycopodiella cernua* (L.) Pic. Serm. | hingble | whole plant | T | anti-sorcery, measles, malaria, fever | 433 |
| *Mallotus oppositifolius* (Geiseler) Müll.Arg. | gbenoukan (F), tchnetchne (G), ayaja (T) | leaves, root, bark | HB, T, EA | CBD atita, asthma, meconium removal, teething | 243, 436, 452 |
| *Mangifera indica* L. | amangua houhou (F, N, Y), mangue (Fr) | leaves, bark | T, HB | respiratory problems, fever, strengthener, newborn strength | NC |
| *Manihot esculenta* Crantz | manioc (Fr) | root | D | malaria | NC |
| *Melaleuca leucadendra* (L.) L. | bpema (F) | leaves | HB | malaria | NC |
| *Merremia tridentata* (L.) Hallier f. | abibey (A), tama (F), fakale (G) | leaves, whole plant | T, HB | diarrhea, sores, CBD atita, fontanels, CBD ka | 212, 364, 421, 432 |
| *Milicia excelsa* (Welw.) C.C.Berg | loko (A, F) | exudate | EA | fontanels | NC |
| *Millettia thonningii* (Schum. & Thonn.) Baker | assousouman (F) | leaves | T | malaria | NC |
| *Mimosa* cf. *quadrivalvis var. leptocarpa* (DC.) Barneby | boassaman (F) | leaves | T | fontanels | NC |
| *Momordica balsamina* L. | kpalari (N) | leaves | T, HB | measles | NC |
| *Momordica charantia* L. | yinsikin (F), assossikan (G), tchati (T) | whole plant, leaves | T, HB, A, EA, E | measles, diarrhea, CBD atita, fever, constipation, antibiotic | 149, 254, 409, 525 |
| *Monodora* cf. *tenuifolia* Benth. | sonoufoko (F, G) | seeds | T, HB | walk early, measles | NC |
| *Monodora myristica* (Gaertn.) Dunal | sassalinkoun (F) | seeds | EA, T | umbilical cord, post-circumcision, constipation, intestinal cleanse, toothache, preventative | NC |
| *Morinda lucida* Benth*.* | atikeysibey (A), honswuey (K), kwenso (M) | leaves | HB, T, D | convulsions, constipation, intestinal cleanse, fever | 133, 365, 537 |
| *Moringa oleifera* Lam. | kpayêdêdê (F), kpatinman (F, G), batamavi (K) | leaves | DR, T, HB, D | headache, diarrhea, fever, headache, anti-sorcery | NC |
| *Mucuna* cf. sp. | feman (F) | leaves | HB | newborn strength | NC |
| *Mucuna pruriens* (L.) DC. | dukey (A), ewe agbakila (N) | leaves | A, HB, T | measles, diarrhea | 488, 450 |
| *Musa* sp*.* | banane (Fr) | leaves | T, D | strengthener, convulsions | NC |
| *Newbouldia laevis* (P.Beauv.) Seem. | adama (F), akokoun (F, G, T), desey sigema (M) | leaves, seeds | EA, T, D, HB | post-circumcision, constipation, malaria, newborn strength, fever, anti-sorcery, | 279 |
| *Nicotiana tabacum* L. | azoman (F), taba (N), ayureawe (T) | leaves | T, EA | malaria, convulsions, umbilical cord, fontanels | NC |
| *Ocimum americanum* L. | hisihisi (F), fio (G) | leaves, whole plant | EA, T, HB, EN, | sore throats, wounds, asthma, cough, fever, post-circumcision, CBD atita, constipation, meconium removal, newborn strength, diarrhea, fontanels, CBD ka, measles, strengthener | 544 |
| *Ocimum basilicum* L. | akohoun (F, G, Y) | leaves | T, D | constipation | NC |
| *Ocimum gratissimum* L. | tchayo (F, G, N), koumoba (T) | leaves, whole plant | T, HB, EA, E, EN | post-circumcision, CBD atita, premature birth, convulsions, walk early, asthma, cough, enema, intestinal cleanse, constipation, meconium removal | 272, 498 |
| *Ocimum* sp. | kessou-kessou (F, G) | whole plant, leaves | T, D, HB | antibiotic, constipation, anti-sorcery, diarrhea, post-circumcision, malaria, newborn strength, fever | NC |
| *Olax subscorpioidea*  Oliv. | mitindo (F) | root | T | intestinal cleanse, constipation | NC |
| *Oldenlandia* cf. *affinis* (Roem. & Schult.) DC. | ahonman (F, G) | leaves | EA, HB | fontanels, premature birth | NC |
| *Opuntia* sp. | cactus (Fr) | root, leaves | T | cough | NC |
| *Pancratium trianthum* Herb. | kouyoman (F, G) | leaves, stem | T | asthma, cough, anti-sorcery | 210, 422 |
| *Parkia biglobosa* (Jacq.) G.Don | awe (F, G), igba (T) | branch, bark, leaves | HB, T | strengthener, constipation, respiratory problems, convulsions, diarrhea, walk early, anti-sorcery, measles | NC |
| *Passiflora foetida* L. | avounyinmitoé (F) | whole plant | T | CBD ka | NC |
| *Paullinia pinnata* L. | ahichan (A), hedoulifi/lokoman (F, G), ganganizema (M) | leaves, root | HB, T | diarrhea, newborn strength, convulsions, post-circumcision, cough | 114, 146 |
| *Pavetta* cf. *crassipes* K.Schum. | gongwako (T) | leaves | T | malaria | NC |
| *Pavetta corymbosa* (DC.) F.N.Williams | lohoun (F) | leaves | T | malaria, newborn strength | NC |
| *Pennisetum* cf. *glaucum* (L.) R.Br. | mil (Fr) | seeds | E | measles | NC |
| *Pergularia daemia* (Forssk.) Chiov. | bonukeykey (A), awinikunsiewa (M) | leaves | EA | fontanels, cough | 493 |
| *Periploca calophylla* (Baill.) Roberty | homa/asobokan (F) | leaves | EA | newborn strength, umbilical cord | 475, 583, 608 |
| *Persea americana* Mil. | avocamanhouhou (F) | leaves | D | asthma | NC |
| *Phyllanthus amarus* Schumach. & Thonn. | hlinwhé (F), tehisso (N, Y), aibiso (T) | whole plant, leaves | T | constipation, meconium removal, malaria, newborn strength, intestinal cleanse, vermifuge, diarrhea | NC |
| *Physalis* cf. *angulata* L. | korogba (F, N), kongba (Y) | whole plant | T, HB | constipation, CBD ka, CBD atita, measles | NC |
| *Piper guineense* Schumach. & Thonn. | pimment du guinea (Fr), injaive (T) | fruit | DR, T, D, EA, HB | headache, CBD ka, CBD atita, fontanels, asthma, strengthener, constipation | 658 |
| *Plectranthus monostachyus* (P.Beauv.) B.J.Pollard | koumoba (T) | leaves | T | constipation | 242 |
| *Portulaca grandiflora* Hook. | dri (G) | whole plant | T | teething | NC |
| *Prosopis africana* (Guill. & Perr.) Taub. | kakè (F, G) | wood | HB, T | newborn strength, walk early, constipation, fever | 401 |
| *Pseudocedrela* cf. *kotschyi* (Schweinf.) Harms | tchagigi (T) | leaves | T | vermifuge | NC |
| *Psidium guajave* L. | kinkouman (F, G, N) | leaves | T, EA, D | diarrhea, post-circumcision, asthma | NC |
| *Psychotria psychotrioides* (DC.) Roberty | atindohoussa (F) | bark | HB | post-circumcision | NC |
| *Psychotria vogeliana* Benth. | deblago (G) | leaves | T | post-circumcision | 414 |
| *Pteleopsis suberosa* Engl. & Diels | kulikuligoto (F) | bark, leaves | T, HB | CBD atita, CBD ka, newborn strength, measles | NC |
| *Pterocarpus erinaceus* Poir. | kosso (G) | bark | T | walk early | NC |
| *Pterocarpus santalinoides* DC. | gbengbè (F, G), begbema (M) | leaves | T, HB, D | diarrhea, newborn strength, constipation, CBD atita | 138, 634 |
| *Pupalia lappacea* (L.) Juss. | tredoagbokokui (F) | seeds | EA | fontanels | 455 |
| *Pycnanthus* cf. *angolensis* (Welw.) Warb. | yaya (F) | leaves | T | asthma, cough | NC |
| *Raphia hookeri* G. Mann & H. Wendl. | dekui/alitadekoun (F, G) | seeds | T, HB | fontanels | NC |
| *Raphia* sp. | ramo (F) | leaves | C | anti-sorcery | NC |
| *Rauvolfia vomitoria* Afzel. | vonmansi (G) | leaves | HB, T | fontanels, fever | 514 |
| *Remirea maritima* Aubl. | houyin/housso (F, G) | whole plant | T | teething | 417 |
| *Rhaphiostylis beninensis* (Hook.f. ex Planch.) Planch. ex Benth. | gbagblakan (F, G) | wood | T | newborn strength, constipation, intestinal cleanse | NC |
| *Rhodognaphalon* cf. *brevicuspe* (Sprague) Roberty | patindeyhun (F) | leaves | EA | rib/bone displacement | NC |
| *Ricinus communis* L. | fefekoupa (T) | leaves | SB, D | fever, stomachache | NC |
| *Rourea coccinea* (Schumach. & Thonn.) Benth. | vikplonbaman (G), amedje (N) | leaves | T | post-circumcision, diarrhea | 441 |
| *Rytigynia senegalensis* Blume | gbadema (F, N, Y) | leaves | T, HB | malaria, measles | 411 |
| *Sansevieria liberica* Gérôme & Labroy | kpohiando/kponman (F) | root, leaves | T | malaria, fever | NC |
| *Sarcocephalus latifolius* (Sm.) E.A.Bruce | kudo (F), umbesi (T) | root | T | intestinal cleanse, constipation, malaria | NC |
| *Schrebera arborea* A.Chev. | fadou (F) | seeds | HB | fontanels | 407 |
| *Schwenckia americana* L. | amankoukui (F) | leaves, whole plant | HB, T | post-circumcision, measles, CBD atita, fontanels | NC |
| *Secamone afzelii* (Roem. & Schult.) K.Schum. | zungikusi (F), zougoudou (G), ablengblo (T) | leaves | T, HB, EA, E | convulsions, CBD ka, fontanels, constipation, intestinal cleanse, CBD atita, cough | 299, 597 |
| *Securidaca* cf. *longipedunculata* Fresen. | patado (F) | root | T | asthma, cough | NC |
| *Senna alata* (L.) Roxb. | amasou (F), dumadòsogomè (K) | leaves | T, D | intestinal cleanse, constipation, meconium removal | 518 |
| *Senna hirsuta* (L.) H.S.Irwin & Barneby | batomayi (F) | leaves | HB | fever | 471 |
| *Senna obtusifolia* (L.) H.S.Irwin & Barneby | kpanhouman (F) | leaves | EA | umbilical cord, wounds | 507 |
| *Senna occidentalis* (L.) Link | agolikan (F), anajabulo (T) | leaves | T, EA, HB | diarrhea, fontanels, newborn strength, strengthener, convulsions, crisis, fever, malaria | 241, 600 |
| *Senna siamea* (Lam.) H.S.Irwin & Barneby | zangla/cassia (A, F), acacia (G, T) | leaves | T, HB | malaria, constipation | 273, 459 |
| *Sesamum indicum* L. | sesame (Fr) | seeds | E | teething | NC |
| *Sida* cf. *rhombifolia* L. | ghema (M) | leaves | EA | toothache | NC |
| *Solanum aethiopicum* L. | gboman/gble (F) | leaves | T, HB | diarrhea, post-circumcision | 598 |
| *Solanum americanum* Mill. | moru (T) | leaves | D | cough | 262 |
| *Solanum dasyphyllum* Schumach. & Thonn. | irwawaudi (T) | leaves | HB | teething | 240 |
| *Solanum lycopersicum* Lam. | tomati (F) | leaves | EA | measles, infections, abscesses | NC |
| *Sorghum bicolor* (L.) Moench | adako (F) | leaves | T | toothache | NC |
| *Sorghum* sp. | hokoveman (G) | leaves | HB | strengthener | 438 |
| *Spondias mombin* L. | akinkoma (F), djogbeman (G) | leaves | T | diarrhea, teething | 440, 635 |
| *Stachytarpheta cayennensis* (Rich.) Vahl | alotrohe (G) | whole plant | HB | premature birth | NC |
| *Stipularia africana* P.Beauv. | towedo (F, G) | root, leaves | T | cough, convulsions, malaria, fever | 424 |
| *Strophanthus hispidus* DC. | afeyfey (T) | leaves | HB | malaria | 358 |
| *Strophanthus* sp. | tegbesu (F) | leaves | E, T | convulsions | 588 |
| *Stylosanthes erecta* P.Beauv. | aduma (A, F) | whole plant, leaves | HB, T | teething | 464 |
| *Syzygium aromaticum* (L.) Merr. & L.M.Perry | atinkenbodata (F, G, M, N) | flower buds, bark | D, HB, T, EA | constipation, newborn strength, post-circumcision, umbilical cord, intestinal cleanse, preventative | NC |
| *Syzygium guineense* (Willd.) DC. | mlanmi (G) | leaves | T | CBD atita | 431 |
| *Tectona grandis* L.f. | teekma (F, M) | leaves | HB | newborn strength | NC |
| *Terminalia glaucescens* Planch. ex Benth. | alotou (F), aloaton (G) | root | T, HB | asthma, cough, post-circumcision, CBD atita, anti-anti-sorcery | NC |
| *Tetrapleura tetraptera* (Schum. & Thonn.) Taub. | lindja (F) | fruit | T | asthma | NC |
| *Thonningia sanguinea* Vahl | atinmahudè (F, G), oyo (N, Y) | whole plant | T | respiratory problems, asthma, cough, constipation, teething | NC |
| *Tithonia diversifolia* (Hemsl.) A.Gray | botowo | leaves | EN, HB | convulsions | 278 |
| *Trema orientalis* (L.) Blume | afere (N, T, Y) | leaves | T, SB | walk early, fever | 258 |
| *Tribulus terrestris* L. | gendarme (F) | whole plant | T | teething | 420 |
| *Tridax procumbens* (L.) L. | kpokpo (G, N, Y) | whole plant | A, T, HB | convulsions, strengthener | 478 |
| *Triumfetta rhomboidea* Jacq. | adjatou (F) | leaves | EA | post-circumcision | NC |
| *Uraria picta* (Jacq.) DC. | asoinsoin (F) | leaves | T | malaria, respiratory problems, asthma, cough | 416 |
| *Utricularia* cf. *spiralis* Sm. | kologakolesi (T) | leaves | HB | fetus strengthener | NC |
| *Uvaria chamae* P.Beauv. | aylahado/aylahaman (F,G, M, N) | root, leaves | T | convulsions, constipation, CBD atita, asthma, malaria, fever, newborn strength | NC |
| *Vepris* cf. *verdoorniana* (Exell & Mendonça) Mziray | akode (F,G) | leaves | T | malaria, constipation, intestinal cleanse | NC |
| *Vitellaria paradoxa* C.F.Gaertn. | limangoto (F,G), beur de karite (Fr) | seeds, bark | T, EA | cough, respiratory problems, diarrhea | NC |
| *Waltheria indica* L. | avoudido (F), avidido (G) | root, whole plant | T | convulsions | 423 |
| *Xylopia aethiopica* (Dunal) A.Rich. | kpedjre (F, G, M, N), arn (T) | fruit, bark | EA, D, T, HB, SB | umbilical cord, intestinal cleanse, constipation, post-circumcision, malaria, toothache, diarrhea, meconium removal, stomachache, fever, strength | NC |
| *Zanthoxylum* sp. | atchanhanwou (F), heja (M) | leaves | T | diarrhea, intestinal cleanse, constipation | 147, 457 |
| *Zanthoxylum zanthoxyloides* (Lam.) Zepern. & Timler | hedou (F) | root | T | diarrhea | NC |
| *Zapoteca portoricensis* (Jacq.) H.M.Hern. | azonkidjado (F), ingbanu (T) | root | EN, T | intestinal cleanse, convulsions | 245 |
| *Zea mays* L. | mais (Fr) | fruit | T, E | diarrhea, CBD ka, measles | NC |
| *Zingiber officinale* Roscoe | dote (F), gingembre (Fr), atalye (T) | rhizome | T, EA, EN, D | asthma, fontanels, intestinal cleanse, constipation | NC |

^a^ Local languages are abbreviated: (A)= Adja; (F)= Fon; (Fr)= French; (G)= Goun; (K)= Kotafon; (M)= Mina; (N)= Nago; (T)= Tcha; (Y)= Yoruba.

^b^ Preparations are abbreviated: (A)= soaked in alcohol; (AT) attach; (C)= ceremony; (D)= drink; (DR)= drops; (E)= eat; (EA)= external application; (EN) = enema;

(HB)= herbal bath; (M)= massage; (SB)= steam bath; (T)= tea

^c^ Use category abbreviations are as follows: CBD= cultural bound disease

^d^ Botanical voucher number and collector initials; NC= not collected.
