# Supplementary material for: Traditional Medicine and Childcare in Western Africa: Mothers’ Knowledge, Folk Illnesses, and Patterns of Healthcare-Seeking Behavior
Source: PLoS One. 2014 Aug 22;9(8):e105972. doi: 10.1371/journal.pone.0105972 (PMC4141852; doi:10.1371/journal.pone.0105972)
Supplement: Table S2 — Species cited in 38 questionnaires in Gabon: scientific botanical name, vernacular plant name(s), plant part used, preparation, use category and collection number. (DOCX) [file pone.0105972.s002.docx]

**Table S2**

Species cited in 38 questionnaires in Gabon: scientific botanical name, vernacular plant name(s), plant part used, preparation, use category and collection number

| Botanical Name | Vernacular Name^a^ | Used part | Preparation^b^ | Use category^c^ | AMT # |
| --- | --- | --- | --- | --- | --- |
| *Acalypha paniculata* Miq. | oekoenkoenakoen (F) | leaves, bark | A | CBD onkoe abijel | NC |
| *Acanthus montanus* (Nees) T.Anderson | ndu (F) | leaves | D | cough | NC |
| *Acmella caulirhiza* Delile | andongsie/andusi (F) | leaves | DR | fontanels | 759, 856 |
| *Aframomum citratum* (C.Pereira) K.Schum. | adzom (F) | herb | DR, D | umbilical cord, malaria, CBD la rate | NC |
| *Aframomum giganteum* (Oliv. & D.Hanb.) K.Schum. | obadzom (F) | leaves | HB | respiratory problems | NC |
| *Aframomum melegueta* K.Schum | ondodo/ondon/ndong (F), petite pimment (Fr) | fruit | EN, T, S, E, EA | intestinal cleanse, flu, fontanels, post-circumcision, fontanels | 780, 1060, 1275 |
| *Aframomum* sp. | adzom ebaja/ajom/bisom/eson (F), petite pimment rouge/piment indigiene (Fr) | fruit, leaves, root | EA, M, V, SiB, D, E, EN, HB | fontanels, CBD fesse rouge, fever, CBD la rate, post-circumcision, flu, malaria, CBD pogha, fetus strengthener | 1088, 1089 |
| *Ageratum conyzoides* (L.) L. | ikukwey(F), hedilikii/hedoki/kombavingi (M), etombijoro (Om), mambi matap/manmibatabe (P) | leaves, root | D, EN, HB | diarrhea, fever, CBD ebem, cough, fever | 1054, 1318 |
| *Albizia* sp. | vovo-esak (F) | whole plant | EN | diarrhea | 1228 |
| *Alchornea cordifolia* (Schumach. & Thonn.) Müll.Arg. | agbuin/nkabi/unsusum abui (F) | leaves | DR, T, SB, EN | toothache, malaria, CBD fesse rouge | 1062, 1231 |
| *Alchornea floribunda* Müll.Arg | alan/alan-bwikili (F) | leaves, root | HB, EN | earache, umbilical cord, intestinal cleanse | 1057, 1200 |
| *Allium cepa* L. | onion (Fr) | stem | M | teething | NC |
| *Alstonia boonei* De Wild. | ekouk/kenkina (F) | bark | D, T, E, EN | asthma, malaria, CBD la rate, vermifuge | 855 |
| *Alstonia* sp. | ekok (F) | bark | D | vermifuge | 809 |
| Amaryllidaceae sp. | molo mundju (F) | leaves | EA | walk early | NC |
| AMT 1087 | inziga kusu (F) | liana | D | lung cleanse | 1087 |
| AMT 1243 | anyang (F) | whole plant | SB | premature birth | 1243 |
| *Annickia affinis* (Exell) Versteegh & Sosef | onvolle/nfo/nfol (F), bois jaune (Fr), mukoka (M), muamba pient (P) | bark, leaves | D, T, E, EA | malaria, fetus strengthener, teething, intestinal cleanse, vermifuge, CBD fesse rouge | 1099 |
| *Annona muricata* L. | corosolle (F), corossolier (Fr) | leaves, bark | SB, D, EN | fever, respiratory problems, fetus strengthener | 871 |
| *Anonidium mannii* (Oliv.) Engl. & Diels | ebom (F) | bark | EN, M | fetus strengthener | NC |
| *Anthocleista* cf. *schweinfurthii* Gilg | ajinebe (F) | bark | D, A | diarrhea, CBD onkoe abijel | NC |
| *Arachis hypogaea* L. | eba-owun (F), huile d'arachide (Fr) | seed | EA | post-circumcision, fontanels | NC |
| *Asparagus warneckei* (Engl.) Hutch. | mincoga mikou (F) | root | DR | fontanels | 864 |
| *Aucoumea klaineana* Pierre | okume/torche indigiene (Fr), okeymone (Om) | bark, resin | D, E, C | diarrhea, anti-sorcery, sores | NC |
| *Baillonella toxisperma* Pierre | oabi (B), azap (F), moabi (Fr), oabey (Os) | bark, leaves | T, EN, SiB, HB | CBD fesse rouge, post-circumcision, fever | NC |
| *Bambusa vulgaris* Schrad | muguisa | bark | EN | CBD fesse rouge | NC |
| *Barteria fistulosa* Mast. | boviongo (B), engokom/ensangom/nsabre (F) | bark | EN, M, T | walk early, fontanels, respiratory problems | 837, 1066, 1303 |
| *Berlinia bracteosa* Benth. | etodo/eybiara (M) | bark | D | intestinal cleanse | NC |
| *Bidens pilosa* L. | biele/oyilee (Ob) | leaves | EA | walk early | 1154, 1173 |
| *Boerhavia diffusa* L. | katakala (Ob) | root | EN | intestinal cleanse | 1170 |
| *Brideli atroviridis* Müll.Arg. | monyombo (B) | bark | T | cough | 1269 |
| *Brillantaisia lancifolia* Lindau | ndolo (F) | whole plant | D | asthma | 1252 |
| *Camptostylus mannii* (Oliv.) Gilg | abumbu/ebubun/ebubung (F) | leaves | HB, A, M, D, EN | respiratory problems, colic, intestinal cleanse, malaria, meconium removal | 852 |
| *Canna indica* L. | ekwanzo (F) | leaves | EC | malaria | 1061 |
| *Capsicum annuum* L | oendodo/okam (F), petite piment/piment/piment rouge (Fr) | leaves, fruit | EN | colic, CBD fesse rouge, diarrhea, meconium removal, CBD la rate, respiratory problems, fontanels, intestinal cleanse | 806 |
| *Carica papaya* L. | papaya (Fr) | leaves, root | T, SB | malaria, fever | 988 |
| *Carpolobia alba* G.Don | onong (F) | root, leaves | HB, D, S, EC | umbilical cord, fetus strengthener, post-circumcision, cough, CBD la rate, malaria, respiratory problems | 779, 1056, 1255 |
| *Carpolobia* sp. | enong (F) | root | S | post-circumcision | NC |
| *Cecropia* *peltata* L. | assong/asung (F) | bark | EN, D | walk early, meconium removal, cold | 1074 |
| *Ceiba* cf. *pentandra* (L.) Gaertn. | guna (M) | bark | E, D | asthma, CBD la rate | NC |
| *Chaetocarpus africanus* Pax | otikancha (Ob) | leaves | EA | fontanels | 1157 |
| *Cissus* cf. *aralioides* (Welw. ex Baker) Planch. | ngun-ele (F) | leaves | EA | fontanels | NC |
| *Cissus* cf. *dewevrei* De Wild. & T.Durand | otoektoek (F) | leaves | EA | CBD fesse rouge | 860 |
| *Citrus aurantiifolia* (Christm.) Swingle | alas/olass (F), citron (Fr) | fruit, leaves | D, EN, HB, SB, T, EA, E | malaria, measles, chicken pox, cough, newborn health, asthma, stomachache, CBD la rate, constipation, meconium removal, CBD pogha, CBD fesse rouge, diarrhea, | 1059, 1168 |
| *Citrus* sp. *CF* | canne acid (Fr) |  | HB | measles | NC |
| *Cleisthopholis* sp. *CF* | avum (F) | bark | DR | newborn health | NC |
| *Clerondendron* sp. | bejim elok/beyemalol/ebele bejium (F), reine des herbes (Fr) | leaves | EA, A, M | fontanels, CBD onkoe | 851 |
| *Coffea canephora* Pierre ex A.Froehner | café (Fr) | leaves | SB | fever | 1158 |
| *Cogniauxia podolaena* Baill. |  | root | DR | CBD la rate | 1229 |
| *Cola* cf. *digitata* Mast. | hekoa (M) | bark | HB | measles | NC |
| *Cola* sp. | abe/abu (F), kola (Fr) | seed, fruit, bark | S, D, E, EA | post-circumcision, cough, fetus strengthener, fontanels | 874 |
| *Colocasia esculenta* (L.) Schott | taro (M) | bark | D | CBD la rate | NC |
| *Combretum aphanopetalum* Engl. & Diels | sissa (F) | leaves | EA | CBD fesse rouge | 859 |
| *Costus ligularis* Baker | mukusa rouge (B) | whole plant | T | cough | 1297 |
| *Costus* sp. | myen (F), canne sauvage (Fr), obong (T) | leaves, stems, whole plant | D, EN, M, EA, SiB, EN, HB, DR, T | asthma, diarrhea, malaria, CBD fesse rouge, CBD la rate, measles, fontanels, CBD bad lungs after birth, chicken pox, fever, respiratory problems | 987 |
| *Coula edulis* Baill. | ohoungou (M) | bark | E | diarrhea | NC |
| *Croton* cf. *oligandrus* Pierre ex Hutch | obumba |  | EN | CBD la rate | NC |
| *Croton mayumbensis* J.Léonard | dibamba (B) | bark | HB | fetus strengthener, CBD pogha | 1264 |
| *Cucumeropsis mannii* Naudin | inchoko/jokou (B), concombre traditionelle (Fr), joka (Ob) | seed, stem, leaves | E, V, DR, HB | diarrhea, walk early, growth stimulation, cough, fetus strengthener | NC |
| *Cyathula prostrata* (L.) Blume | chatee (B), kolo/kolok (F), oborbe grande feuille (Fr) | leaves, seeds, flowers | E, EA | diarrhea, fontanels | 831, 893, 1294 |
| *Cylicodiscus gabunensis* Harms | odouma (B), edum (F) | bark | D, HB, EN | malaria, vermifuge | 1301 |
| *Cymbopogon citratus* (DC.) Stapf | tisane (F) | leaves | T, EN | malaria, measles | NC |
| *Cymbopogon* sp. | citronelle (Fr) | leaves | D, T, SB | malaria | NC |
| *Dacryodes* cf.sp. | unguu (B), ebo (C) | bark | E | diarrhea | 1278 |
| *Daniellia klainei* A.Chev. | owengey (B) | bark | HB | CBD pogha | 1280 |
| *Desmodium adscendens* (Sw.) DC. | oborbe petit feuille (Fr) | leaves | EA | fontanels | 833 |
| *Diodella scandens* (Sw.) Bacigalupo & E.L.Cabra | oyemze (F) | leaves | EA, E | toothache, cough | 886, 1234 |
| *Dioscorea bulbifera* L. | bibuma abang (F) | tuber |  | malaria | 1207 |
| *Dioscorea* sp. | nyam (F) | tuber | D | CBD la rate | NC |
| *Distemonanthus benthamianus* Baill | eyem (F) | bark, leaves | HB, D, E | newborn health, fetus strengthener, fontanels | NC |
| *Dracaena fragrans* (L.) Ker Gawl. | alen-okpo (F) | bark | EN | walk early | 1235 |
| *Duboscia* cf. *macrocarpa* Bocq. | abak (F) | leaves | EN | walk early | NC |
| *Dysphania ambrosioides (*L.) Mosyakin & Clemants | ontchoutchoulou (Ob) | whole plant | SB | malaria | 1175 |
| *Eclipta prostrata* (L.) L. | ivainamoye (B), movindera/oyira (Os) | whole plant, leaves | EA, M | CBD fesse rouge, fever, hemorrhoids | 1167, 1273, 1403 |
| *Elaeis guineensis* Jacq. | esong/onbonmiban (F), huile de palme (Fr) | heart, fruit, seeds | E, EA, M | fetus strengthener, fontanels, CBD la rate, heat rash, post-circumcision, sores, intestinal cleanse, meconium removal, umbilical cord, CBD fesse rouge, measles | NC |
| *Eleusine indica* (L.) Gaertn. | alekinedou (Om) | root | M | CBD la rate | 1164 |
| *Emilia coccinea* (Sims) G.Don | mungusungusu (B), alanopo/olonvoe (F) | leaves, whole plant | D, EC, HB, EA | measles, newborn health, umbilical cord, walk early, CBD fesse rouge, meconium removal | 1247, 1285 |
| *Erythrina droogmansiana*De Wild. & T.Durand | esoesoek/esok (F) | bark | M, EN, D, C | CBD la rate, anti-sorcery | 881 |
| *Ficus exasperata* Vahl | ako (F) | bark | EA, D | umbilical cord, cough, fetus strengthener | 1239 |
| *Ficus mucuso* Welw. ex Ficalho | ekoko/ekokok (F) | leaves | EN | colic, meconium removal | NC |
| *Fleroya* cf. *ledermannii* (K.Krause) Y.F.Deng | otzeyzam (F) | bark | M | CBD bad lungs | NC |
| *Geophila afzelii* Hiern | koudou/kudu (B) | whole plant, leaves | V, EA | CBD la rate | 1308 |
| *Gossypium barbadense* L. | coton (F) | leaves | D | asthma | NC |
| *Guibourtia tessmannii* (Harms) J.Leonard | oveng (F) | bark, resin | HB, C | newborn health, anti-sorcery | NC |
| *Harungana madagascariensis* Lam. ex Poir. | atuin (F) | bark, leaves | SiB, EN, DR, HB, | post-circumcision, diarrhea, CBD fesse rouge, post-circumcision, intestinal cleanse, CBD la rate, measles | 778 |
| *Heterotis decumbens* Jacq.-Fél. | sangancho (Om) | leaves | T | malaria | 1166 |
| *Hibiscus acetosella* Welw. ex Hiern | esang (F), l'oseille (Fr) | flower | M | CBD fesse rouge | 885 |
| *Hibiscus* sp. | osaill (F), ozai (Os) | leaves, whole plant | EA, V | fontanels, walk early | NC |
| *Irvingia gabonensis* (Aubry-Lecomte ex O'Rorke) Baill. | mangue sauvage (Fr), muebe (P) | bark, leaves | M, D | CBD la rate, fetus strengthener, fever | NC |
| *Jatropha gossypiifolia* L. |  | leaves | T | asthma | 1159 |
| *Kalanchoe crenata* (Andrews) Haw. | ivivuma (F), odiokiya (M), landunga (Ob), edokia (Os), majujuga (P), jejoujuga, jewa (T) | leaves, whole plant | D, DR, EA | respiratory problems, cold, ear disorders, umbilical cord, antibiotic, cough, flu | 758, 979 |
| *Lantana camara* L. |  | leaves | T | malaria | 1188 |
| *Laporteaaestuans* (L.) Chew | tak-akun (F) | whole plant | SB | premature birth | 1244 |
| *Lasianthera africana* P.Beauv. | mundungu (B) | bark | HB | growth stimulation, strengthen fetus | NC |
| *Leea guineense* G.Don | mbala (Om) | bark | D | fetus strengthener | NC |
| Leguminosae cf. sp. | rekoa (B) | bark | D | malaria | 1265 |
| *Leptactina mannii* Hook.f. | ewas wasakulu (F), bois des os (Fr) | bark, leaves | HB, D | growth stimulation | 814 |
| *Lygodium* cf. *microphyllum* (Cav.) R. Br. | nzalanu (F) | leaves | E | diarrhea | NC |
| *Macaranga barteri* Müll.Arg. | echemey (B) | bark | T | walk early | 1288 |
| *Macaranga saccifera* Pax | mopoapoa (B) | bark | HB | fetus strengthener, CBD pogha | NC |
| *Macaranga spinosa* Müll.Arg | macaranga/mungembe (B), lasass (F) | leaves, bark | EC, EN, T | malaria, walk early, meconium removal, respiratory problems | 1064 |
| *Maesopsis eminii* Engl. | enkangalle (F), mangobey (M) | bark | D, EN, T, HB | cough, CBD fesse rouge, malaria, CBD la rate, CBD pogha | NC |
| *Mangifera indica* L. | endok (F), mangue (Fr) | leaves, bark, root | T, SB, EN, D | malaria, hemorrhoids, diarrhea, fever, CBD fesse rouge | NC |
| *Manihot esculenta* Crantz | menza (F), manioc (Fr), ayaga (Ob) | leaves, tuber | EA, HB, D, EN, | measles, chicken pox, fetus strengthener, intestinal cleanse | NC |
| *Maprounea membranacea* Pax & K.Hoffm. | baobao (Fr) | leaves | D | walk early | 1348 |
| *Melia azedarach* L. | kadunga (Ob) | leaves | T | malaria | 1169 |
| *Millettia gagnepainiana* Dunn | fe-enziee (F) | liana | HB | umbilical cord | NC |
| *Millettia mannii* Baker | diperie (M), vinekwey (F) | bark, liana | D, A | intestinal cleanse, meconium removal, newborn | NC |
| *Mimosa* cf. *diplotricha* Sauvalle | ebata (B) | leaves | V | respiratory problems | 1196 |
| *Momordica* cf. *foetida* Schumach. | eyenzum (F) | whole plant | EN | CBD ebem | NC |
| *Momordica charantia* L. | mabubulu (M), mabunbula (P) | leaves | D, EN, HB | colic, diarrhea, intestinal cleanse, crisis, measles, intestinal cleanse | NC |
| *Morinda lucida* Benth. | akong (F) | bark | D, EN, T, HB | intestinal cleanse, CBD la rate, malaria | 858, 1213, 1214 |
| *Musa* sp. | banna (B), anginve/atoran/elat- onton-ekon/enbok-ono (F), bannane (Fr), makokodo (Os) | leaves, fruit, root | EN, M, D, EA, HB, SB, T, E, A | convulsions, CBD fesse rouge, CBD la rate, cough, umbilical cord, fetus strengthener, meconium removal, malaria, diarrhea, post-circumcision, fontanels, vermifuge | NC |
| *Musanga cecropioides* R.Br. ex Tedlie | enseng (F) | bark | EN | CBD ebem | NC |
| *Myrianthus arboreus* P.Beauv. | angokon/ekokom/enkokun-mieng (F) | bark, fruit, leaves | EA, DR, E, D, EN, EC | fontanels, food, fetus strengthener, walk early, diarrhea, malaria | NC |
| *Myrianthus serratus* (Trécul) Benth. | afulum (F) | whole plant | HB | newborn health | 1251 |
| *Newbouldia laevis* (P.Beauv.) Seem. | l'izop (Fr), ovendo (Om), | bark, leaves | D, HB | cough, good luck | 1187 |
| *Nicotiana tabacum* L. | tabac (Fr) | leaves | EA, M, EN | CBD fesse rouge | NC |
| *Nymphaea lotus* L. | otoeto (F) | leaves | EN | respiratory problems | NC |
| *Ocimum americanum* L. | ocim (F) | whole plant, leaves | DR, EA | earache, walk early | NC |
| *Ocimum gratissimum* L. | massep (F), aduma duma (Ob) | whole plant, leaves | T, EA, D, EN, M, HB | cold, cough, fever, toothache, CBD fesse rouge, diarrhea, intestinal cleanse, umbilical cord | 1072, 1160, 1172 |
| *Ocimum* sp. | ndzip (F), ndiandzi (P) | leaves, whole plant | D, EN | cough, malaria, intestinal cleanse | NC |
| *Oryza sativa* L. | riz (Fr) | seed | E | diarrhea | NC |
| *Palisota* cf. sp. | injokou (B) | leaves | HB | growth stimulation | 1290 |
| *Panda oleosa* Pierre | afan (F) | bark | EN | intestinal cleanse | NC |
| *Parinari excelsa* Sabine | otcha (B) | bark | HB | CBD pogha | 1281 |
| *Passiflora foetida* L. | matuka makari (P) | leaves | D | newborn health, diarrhea | NC |
| *Pennisetum* cf. *glaucum* (L.) R.Br. | wunzuku (P) | leaves |  | newborn health | NC |
| *Pentaclethra* cf. *eetveldeana* De Wild. & T.Durand | tzi (F) | whole plant, bark | DR | colic, intestinal cleanse | NC |
| *Pentaclethra macrophylla* Benth. | ebeng/nzesé (F), mpandzi (M), ompie (T) | bark, wood, seeds | M, EN, D | CBD la rate, asthma, fetus strengthener | 834, 1077 |
| *Perichasmalaetificata* Miers | tsigue (F) | leaves, stem | EN, A, D | diarrhea | 810, 832, 863 |
| *Persea americana* Mill. | afia (F) | bark |  | toothache | 1078 |
| *Phaseolus vulgaris* L. | haricot (F) | leaves | D,HB | measles | NC |
| *Phyllanthus amarus* Schumach. & Thonn. | kunguh (F) | whole plant | T | flu | 1053 |
| *Phyllanthus* sp. | kanguh (F) | whole plant | T | diarrhea | 1245 |
| *Picralima nitida* (Stapf) T.Durand & H.Durand | dumavendo (B), ansongomo (F), dirundu (M) | bark | D, EN | asthma, CBD la rate, malaria | NC |
| *Piper umbellatum* L. | abomanzan/obadzom (F), malemto (P), | leaves, whole plant | EA, EN, HB | hemorrhoids, post-circumcision, intestinal cleanse, growth stimulation, measles | 877, 1246 |
| *Piptadeniastrum africanum* (Hook.f.) Brenan | miso-miso/tum (F) | bark | EN | CBD la rate | 816a |
| *Plagiostyles africana* (Müll.Arg.) Prain | esula (F) | bark | HB | newborn health | NC |
| *Portulaca oleracea* L. | afosi (F), oyabi (Ob) | whole plant | SB, E | premature birth, sores | 1176 |
| *Pseudospondias longifolia* Engl. | ofoss (F) | fruit | E | kids' food | 1081 |
| *Psidium guajava* L. | guave (F) | leaves | D, T, SB | diarrhea | NC |
| *Psydrax*cf. *palma* (K.Schum.) Bridson | colera (F) | herb | EA | fontanels | NC |
| *Pterocarpus soyauxii* Taub. | motobo (B), esi/umbey (F), kaolin rouge (Fr), motomba/padouk (M) | wood, bark | SM, S, EA, HB, D, EN | post-circumcision, fontanels, measles, chicken pox, umbilical cord, fontanels, walk early, crisis, respiratory problems, diarrhea | NC |
| *Pycnanthus angolensis* (Welw.) Warb. | ecombo/muchoko (B), etong (F), otchokou (M) | bark, leaves | T, M, D, EN, SiB, HB | respiratory problems, fontanels, excessive salvia, cough, CBD fesse rouge, CBD pogha | 1076, 1090, 1195, 1284 |
| *Quassia* *africana* (Baill.) Baill. | izien iral (M) | root | D | malaria | 895 |
| *Rauvolfia mannii* Stapf | obaton (F) |  | E, D, T | malaria, CBD la rate | NC |
| *Ricinodendron* cf. *heudelotii* (Baill.) Heckel | essessa (F) | leaves | D | fetus strengthener | NC |
| *Saccharum officinarum* L. | enkok (F), canne sucre (Fr) | whole plant, stem | D, T, E | asthma, flu, malaria, meconium removal | NC |
| *Sacoglottis gabonensis* (Baill.) Urb. | ozohgo (B) | bark | EA | CBD la rate | NC |
| *Sarcocephalus latifolius* (Sm.) E.A.Bruce | ebohwey (Os), ondolo (T) | root, bark | D | malaria, anti-sorcery | 1404 |
| *Scleria boivinii* Steud. | zengey (B, M), fofolou (F), laim sauvage (Fr), kengitsie (P) | leaves, whole plant | DR, EA | umbilical cord | 1199 |
| *Scoparia dulcis* L. | mnserè (F), ogandarga (Om) | leaves, whole plant | D, TP | vermifuge, walk early | 830, 1165 |
| *Scorodophloeus zenkeri* Harms | kaakey (B) | bark | HB | CBD pogha, fetus strengthener | NC |
| *Senna alata* (L.) Roxb. | moviovo (B), dowlontou (F), kinkiliba (Fr), kangadiba (M), angare/oumara (T) | leaves | D, EA, EN, T | stomachache, blisters, diarrhea, CBD fesse rouge, malaria, constipation, meconium removal | 1210, 1320 |
| *Senna occidentalis* (L.) Link | besi (F), ngari (Ob) | whole plant, leaves | EN, EA | CBD la rate, skin diseases | 1055 |
| *Sesamum radiatum* Schumach. & Thonn. | mokoka (Os) | leaves | HB | fever | 1405 |
| *Sida acuta* Burm.f. |  | whole plant | M | walk early | NC |
| *Solanecio angulatus* (Vahl) C.Jeffrey | budiambu | leaves | D | crisis | NC |
| *Solanum americanum* Mill. | otchango (M) | leaves | D | cough, fetus strengthener, fever | 1323 |
| *Spathodea* cf. *campanulata* P.Beauv. | evuvum (F) | leaves | DR | cough | NC |
| *Staudtia kamerunensis var. gabonensis*(Warb.) Fouilloy | oghobey (C) | bark |  | cough | 1256 |
| *Streptogyna* cf. *crinita* P.Beauv. | bongi (M) | whole plant | E | diarrhea | NC |
| *Tabernanthe iboga* Baill. | bois sacre (Fr) | root | D | fetus strengthener, anti-sorcery | NC |
| *Telfairia* cf. *pedata* (Sm. ex Sims) Hook. | ayuzum (F) | leaves | D | colic, meconium removal | NC |
| *Terminalia catappa* L. | huile d'almande (Fr) | seed | EA, M | fontanels, post-circumcision, measles, fever, CBD fesse rouge | NC |
| *Tetracera* sp. | nzrnzu (F) | wood | D | fetus strengthener | NC |
| *Tetrapleura* cf. *tetraptera* (Schum. & Thonn.) Taub. | ozara (B) | bark | HB | CBD pogha | 1302 |
| *Tetrorchidium didymostemon* (Baill.) Pax & K.Hoffm. | nzili (F), ngoumou (Ob) | leaves, bark | D, EN | colic, constipation, meconium removal, vermifuge | 1171, 1202 |
| *Thomandersia congolana* De Wild. & T.Durand | umbazal (F) | root | D | fetus strengthener | 1253 |
| *Tithonia diversifolia* (Hemsl.) A.Gray | margarit (F) | leaves, whole plant, flower | EN, EA, HB, M | CBD la rate, intestinal cleanse, malaria, measles, CBD fesse rouge | 862 |
| *Vernonia amygdalina* Delile | bikambilar/joloyolo/zomalyo (F), kongobulubu/ondole (Ob), kungubulu (T) | leaves, bark | EN, D, HB, EC | intestinal cleanse, toothache, vermifuge, CBD la rate, measles, malaria, chicken pox | 807, 980, 1070, 1153, 1174 |
| *Vernonia conferta* Benth. | abanga/abankak (F) | bark | T, D | diarrhea | 1071, 1201 |
| *Vernonia* sp. | mopotopoto (B) | whole plant |  | CBD la rate | 1257 |
| *Vitellaria paradoxa* C.F.Gaertn. | berre de carite (Fr) | seed | M | CBD la rate | NC |
| *Xylopia aethiopica* (Dunal) A.Rich. | bikwin (F) | fruit | S | post-circumcision | NC |
| *Zanthoxylum* cf. *heitzii* (Aubrév. & Pellegr.) P.G.Waterman | olom (F) | bark | D | asthma | NC |
| *Zea mays* L. | mais (Fr) | fruit | EA, HB | measles | NC |
| *Zingiber officinale* Roscoe | gingembre (Fr), maketa (Om) | rhizome | D, EA, EN, V | cough, CBD fesse rouge, respiratory problems | NC |

^a^ Local languages are abbreviated: (B)= Babungu; (C)= Commercial timber name; (F)= Fang;(Fr)= French; (M)= Mitsogo; (Ob)= Obamba; (Om)= Omiene; (Os)= Ossimba; (T)= Teke

^b^ Preparations are abbreviated: (A)= attach; (C)= ceremony; (D)= drink; (DR)= drops; (E)= eat; (EA)= external application; (EC)= encircle; (EN) = enema; (HB)= herbal bath; (S)=

spit; (SB)= steam bath; (SiB)= sit bath; (SM)= envelop in smoke; (T)= tea; (TP)= tap on feet; (M)= massage; (V)= vaccination

^c^ Use category abbreviations are as follows: CBD= cultural bound disease

^d^ Botanical voucher number and collector initials; NC= not collected.
